# **When Health Systems Consider Research to Be Beyond the Scope of Healthcare Delivery, Research Translation Is Crippled** Comment on "Academic Health Science Centres as Vehicles for Knowledge Mobilisation in Australia? A Qualitative Study"

**DOI:** 10.34172/ijhpm.2021.104

**Published:** 2021-08-25

**Authors:** Christine Jorm, Donella Piper

**Affiliations:** School of Medicine and Public Health, The University of Newcastle, Callaghan, NSW, Australia.

**Keywords:** Co-Production, Design Thinking, Impact, Knowledge Mobilisation, Context, Research Funding

## Abstract

Edelman and colleagues’ analysis of the views of Board members of Australian Research Translation Centres (RTCs) is well timed. There has been little study of Australian RTCs to date. We focus on their recommendations regarding knowledge mobilisation (KM) to open broader debate on the wisdom of regarding UK practices as a blueprint. We go further and ask whether successful RTCs might, as a result of responding to local context, create idiosyncratic structures and solutions, making generalisable learning less likely? There has been much invested in Australian RTCs and implications of government’s formative evaluation of their work is discussed. Five recommendations are made that could help RTCs: allowing system end-users a greater say in funding decisions, taking a broader, more democratic approach to kinds of knowledge that are valued; investing in methodologies derived from the innovation space; and, a creative attention to governance to support these ideas.

 Edelman and colleagues interviewed 15 Board members of four academic health science centres in 2019 asking how people, processes and systems are being organised within these centres to enable knowledge to be mobilised for impact.^1^ (The term academic health science centre is not uniformly used and in this commentary we take the approach of other recent publications^2,3^ and use an overarching term for both UK and Australian centres: Research Translation Centres [RTCs]). The Australian RTCs studied had very different contexts; in the nature and number of their partners, the population served, investment made, relationship with State health system managers and in the details of RTC staffing and governance structure.

 This work contributes to the slim research published to date on the Australian RTC experience. While conceding the RTCs were in a formative stage of development the authors critiqued their focus on structure and governance, calling instead for ‘action-oriented roles and processes.’^1^ It was considered that a narrow view of research translation existed despite international experience providing a clear blue-print for ideal knowledge mobilisation (KM) processes. There was also found to be a dynamic tension between collaboration and competition in the academic organisations that had come together in these centres and comment was made that establishment takes time.

## Knowledge Mobilisation – Do We Know Exactly What Should Be Done?

 Knowledge is no longer considered as being generated objectively and at a remove from those who might eventually use it, but instead as constructed from social interaction and negotiation and therefore, dynamic and contested, relational and thus always contextually sensitive.^4^ Mere translation is problematic and highly limited in its effectiveness to drive change:

*“By having deferred engaging with the messiness of reality until it gets to the point of knowledge translation, elegant knowledge has in effect exempted itself from negotiating its contents with end users, from adapting itself to complex situations, from reinventing itself in response to emergent problems and from having to acknowledge that local practices embody their own ecologies and their own wisdoms*.”^5^

 Accepting the broader current understanding, *terms* knowledge translation and KM are still often used interchangeably by many clinicians and managers so it is not entirely clear what significance the use of the older term translation by a Board member, as singled out by the authors, might have. However, authors claimed they also found limited development of KM *processes*, a matter of concern for an RTC. This claim was based on perceived deficits in processes of negotiation and knowledge broker roles:

*“Whilst [ they] described using several strategies to effect research translation in clinical settings, including research capacity building, participatory approaches, flagship projects and clinical engagement, there was little overall mention of processes of negotiation to systematise the utilisation of knowledge*.”^1^

*“… although there was a focus … on research capacity building within clinical settings and mention of goals to co-produce research, there was no specific reference … to establishing knowledge broker or boundary spanner roles, which feature as key knowledge mobilisation processes elsewhere*.”^1^

 Central among the key principles proposed for effective KM is fostering and sustaining relationships between researchers and research users over time (as interactions and collaboration co-produce knowledge).^4,6^ Our own work on embedded economic research^7^ suggests that strong relationships are integral to influence and learning and time is essential to building these.^5^ The approaches reported by RTC Board members: participatory, capacity building, co-production and clinical engagement – are all relationship building.

 There is still a limited evidence base for many common knowledge mobilization interventions including knowledge brokers.^4^ A rapid review of RTCs found issues with such hybrid roles^3^ and respondents to a recent qualitative study of UK and Australian RTCs also reported difficulties.^2^ Instead, collective brokering is now proposed as a more effective alternative to individual knowledge brokers.^8^ The RTC themselves are essentially *brokering structures*,^8^ and if they flourish, designing and undertaking substantive amounts of genuinely collaborative work, KM will follow.

## Formative Learning and Evaluation – Where Should We Look?

 The Australian RTCs studied were young^1^ making the observed focus of interviewees on governance and structures unsurprising: RTCs are complex partnerships. The major body of relevant research relates to the UK Collaborations for Leadership in Applied Health Research and Care (CLAHRCs). These partnerships between universities and the surrounding National Health Service organisations were developed in response to the ‘growing recognition of the need to accelerate the generation and uptake of knowledge in health systems.’^9^ The nine CLAHRC pilots were funded for 5 years from 2014. Their funding was extended and the now 15 are titled ‘National Institute for Health Research (NIHR) Applied Research Collaborations.’

 A systematic review undertaken 6 years post-establishment of the CLAHRCs found most evaluations focused on their organisation and development of theory around their emergent properties.^9^ Evidence was lacking on their impact and little generalisable knowledge had been developed on practices adopted to achieve impact, such as priority setting.^9^ A more recent rapid review of UK and Australian RTC research focused on structures, leadership, workforce development and strategies for involving communities and service users; finding considerable variation.^3^ While there was an agreed need for workforce development, in particular in: leadership, implementation research, KM, evaluation and collaborative priority setting, it was unclear which approaches to development were most beneficial.^3^ Despite years of UK funding, greater structural similarities between UK RTCs compared with Australian RTCs and strong UK health services research, the RTC evidence base remains diffuse.

###  Are RTCs Possibly Organisations, That When Functioning Well, With Diverse Local Partners Collaborating, Create Idiosyncratic Structures and Solutions, Making Meaningful Generalisable Learning Difficult? 

 The Australian National Health and Medical Research Centre (NHMRC) accredited RTCs had anticipated 10 years of funding via the Medical Research Future Fund (MRFF) Rapid Applied Research Translation (RART) grant scheme. This was viewed positively^2^ as UK review had suggested 5 year funding cycles for RTCs as too short.^9^ Edelman and colleagues proffered their recommendations in order to assist with ‘formative learning and evaluation’^1^of Australian RTCs. The Australian government has also recently undertaken a form of formative evaluation via a detailed review of the RART funding scheme.^10^ Only 16%-23% of RART funded projects were complete at the time of review, but the team was concerned about the likelihood of impact ie, significant research translation, finding:

*“There is an opportunity to ensure that … all RART project proposals identify a proposed path to research translation that has been informed by engagement with research end-users and beneficiaries. Those responsible for research adoption should be supportive of the project proposal*.”^10^

 They recommended:

*“…funding research projects that can demonstrate the greatest potential for research translation and adoption in areas of prioritised need. … proposals should provide a plan for research translation which demonstrates commitment from all key research partners*.”^10^

 They also placed importance on ‘*measuring and communicating research progress, outcomes and impact.’*^10^ Following this evaluation major changes were made to the funding scheme. These included the NHMRC accredited RTCs no longer being the only eligible applicants to the scheme – it was made nationally competitive and open to both small scale and larger more complex projects. The new assessment criteria suggest an attempt to reduce focus on researcher-determined priorities and to produce rapid tangible healthcare impact: including requirements to deliver outcomes that are a priority for the Australian public and *within* the grant period. They also required evidence of engagement and involvement by all stakeholders during conceptualisation, development and planned implementation (ie, co-production). The importance of health service delivery partnerships to support rapid implementation into practice was further signalled by requirements for: detailed lists of partner contributions, letters of support and lists of chief investigators and collaborators with shared authority and responsibility for leading and directing the design, conduct and reporting. The latter suggests the possibility of benefit from different approaches to RTC governance such as alliance governance (where shared goals and a systems perspective are given rigor by performance measurement - with a focus on patient outcomes).^11^

## The Way We Fund Health And Medical Research Is Not Helpful to RTCs

 A former head of the NHMRC noted:

*“In most industries, research and development are integral parts of the industry itself. But in health, here and most everywhere, we do something different; we separate research from the delivery of health care*.”^12^

 The results of this situation are evident in both the UK and Australia. The development of the CLAHRCs was influenced by the research metrics required by the NIHR. These included numbers of publications, students awarded higher degrees, additional research funding leveraged as well as case studies demonstrating healthcare impact.^9^ Because of this, many CLAHRCs merely mapped their areas of existing research strength onto the needs of the local health providers.^13^

 The recent UK-Australia RTC qualitative study found:

*“Significant challenges in integrating applied healthcare and improvement approaches with more rigorous discovery and implementation research*.”^2^

 The authors attributed this tension to:* ‘Dissonant metrics between academic and healthcare *sectors.’^2^ Yet awareness of the limited value to society that may be reflected in traditional academic output indicators underlies current interest in research impact assessment. Australian RTC participants identified challenges in ensuring health research was relevant to the needs of front-line staff.^2^ Perhaps the reality of health service needs being not of interest to researchers working to other metrics needs greater attention. A recent academic objection to co-production was blunt:

*“The coproduction process can lead to researchers being asked to answer questions which are dull, not novel (little contribution to the scientific literature), or not generalisable (focused on local issues) – and therefore not easily publishable*.”^14^

 If health and medical research represents research and development (R&D)for the health sector, we are probably also underinvesting. Australia spends approximately 0.3% of gross domestic product on health and medical research, which represents just 3.0% of total health spending.^15^ Despite rising health service demand due to an ageing population and higher incidence of chronic disease, health service research represents under 5% of NHMRC funding (the proportion of MRFF funding dedicated to health service research is as yet unknown but likely to be higher). An appropriate level of investment will only be able to be determined when the health sector has greater control of research funding and is able to focus support on work that meets its priorities.

## Four Proposed Solutions to Support Australian RTCs

 Currently, the RTCs are in an invidious position, by their nature idiosyncratic and emergent, easy to criticise and working within funding systems and incentive schemes (for both academics and health systems) that are set to oppose their ambitions.

1. Allow health services and the community a greater say in decision making at both local and national level in what research is funded.
2. Adopt a more democratic approach to knowledge that is valued.
3. Add methodologies from business and innovation (eg, design thinking) to the work portfolio.
4. Explore governance models that create effective shared responsibility among partners.

The second proposed solution requires a crucial paradigm shift. When researchers seeking improvement use the terms like ‘more rigorous’^2^ they are supporting an ideology that elevates formal research activities above learning from practice and from stakeholders.^5^ The often theoretical and inaccessible research products created then increase system resistance to working with researchers:

*“…such research fails to spend sufficient time on learning … and then elevates the problem of why its advice does not take root in practice as yet requiring more (implementation) ‘science,’ perpetuating the problem on a different front*.”^5^

 We can do better to support Australian RTCs. We must also accept that development of complex organisational partnerships takes time and that co-designed solutions will be context dependent. Generalisable learnings to date are weak and local impact is the major RTC outcome we should be seeking.

## Acknowledgements

 We’d like to thank the two anonymous referees for their thoughtful suggestions which improved the commentary.

## Ethical issues

 Not applicable.

## Competing interests

 CJ was the paid Director of NSW Regional Health Partners, one of Australia’s health RTCs from August 2018 to April 2021 – this paper was written after this and without financial support. DP is employed on a NSW Regional Health Partners MRFF RART grant funded project.

## Authors’ contributions

 CJ: Conception and design. DP: Critical revision of the manuscript for important intellectual content.

## Funding

 DP is supported by an MRFF grant to NSW Regional Health Partners.
